# Trabecular structural difference between the superior and inferior regions of the vertebral body: a cadaveric and clinical study

**DOI:** 10.3389/fendo.2023.1238654

**Published:** 2023-09-19

**Authors:** Dong Eun Shin, Younghun Lee, Hyun-Ju An, Tae-Sun Hwang, Jin-Woo Cho, Jongbeom Oh, Wooyeol Ahn, Jaemin Lee, Chul Gie Hong, Yeonju Lee, Soonchul Lee

**Affiliations:** ^1^ Department of Orthopaedic Surgery, CHA Bundang Medical Center, CHA University School of Medicine, Gyeonggi-do, Republic of Korea; ^2^ SL Bio, Inc., Gyeonggi-do, Republic of Korea; ^3^ Department of Anatomy, School of Medicine, CHA University, Gyeonggi-do, Republic of Korea; ^4^ Department of Orthopedic Surgery, Kangwon National University Hospital, Gangwon-do, Republic of Korea; ^5^ CHA Graduate School of Medicine, Pochon, Republic of Korea

**Keywords:** superior region, inferior region, osteoporosis, compression fracture, bone mineral density, vertebrae

## Abstract

**Background:**

Osteoporotic vertebral compression fractures commonly involve the superior vertebral body; however, their associated causes have not yet been clearly established. This study aimed to determine the trabecular structural differences between the superior and inferior regions of the vertebral body using cadaveric and clinical studies.

**Materials and methods:**

First, five vertebrae were collected from three human cadavers. The trabecular structures of the superior and inferior regions of each vertebral body were analyzed using micro-computed tomography (micro-CT), finite element analysis (FEA), and biomechanical test. Based on the results of the ex vivo study, we conducted a clinical study. Second, spine CT images were retrospectively collected. Bone volume and Hounsfield unit were analyzed for 192 vertebral bodies. Finally, after sample size calculation based on the pilot study, prospectively, 200 participants underwent dual-energy X-ray absorptiometry (DXA) of the lateral spine. The bone mineral densities (BMDs) of the superior and inferior regions of each lumbar vertebral body were measured. The paired t-test and Wilcoxon signed-rank test were used for the statistical analyses, and p-value < 0.05 was considered significant.

**Results:**

Cadaver studies revealed differences between the superior and inferior trabecular bone structures. The bone volume ratio, BMD, and various other trabecular parameters advocated for decreased strength of the superior region. Throughout the biomechanical study, the limitations of the compression force were 3.44 and 4.63 N/m^2^ for the superior and inferior regions, respectively. In the FEA study, the inferior region had a lower average displacement and higher von Mises stress than the superior region. In the clinical spine CT-based bone volume and BMD study, the bone volume was significantly higher in the inferior region than in the superior region. In the lateral spine DXA, the mean BMD of the superior region of vertebral bodies was significantly lower compared with that of the inferior region.

**Conclusion:**

The superior trabecular structure of the lumbar vertebral bodies possesses more biomechanical susceptibility compared with the inferior trabecular structure, confirming its dominant role in causing osteoporotic vertebral fractures. Physicians should also focus on the BMD values of the superior region of the vertebral body using lateral spine DXA to evaluate osteoporosis.

## Introduction

Osteoporosis is a skeletal disease characterized by low bone mass, deterioration of bone tissue, and disruption of bone microarchitecture. Consequently, it can increase in the risk of fractures (1). Osteoporotic vertebral compression fracture (OVCF) is a well-known complication of osteoporosis and causes severe back pain, dysfunction, and high morbidity and mortality rates in elderly patients. In an epidemiological study using nationwide claims data in South Korea, 644,500 OVCF cases were reported from 2012 to 2016. OVCF was most common in patients in their 70s (45%), and the number of patients with OVCF is expected to increase rapidly (2, 3). Interestingly, OVCFs occur most commonly in the thoracolumbar spine, and the superior region of the vertebral body, including the superior endplate and underlying trabeculae, is involved more frequently than the inferior region (4, 5). Of the 211 patients with OVCF, 39% had involvement of the superior endplate, and only 12% had involvement of inferior endplates (4).

Several studies have analyzed the reasons why the inferior endplates in the lower lumbar region may be relatively thickened because of the nutritional demands of the larger discs caudally (6). Moreover, Zhao et al. insisted that the superior endplate was thinner and supported by less-dense trabecular bones than the inferior endplate (5). However, limited information is available regarding the structural differences between the superior and inferior regions of the vertebral body.

Understanding the regional structure is necessary to analyze specific trabecular zones using dual-energy X-ray absorptiometry (DXA) to prevent OVCF. The development of an index for predicting fracture risk may influence decisions regarding patient treatment, playing a major role in the early intervention of vertebral fractures. This study aimed to determine the structural differences between the superior and inferior regions of the vertebral bodies.

## Materials and methods

### Study outline

**Figure 1:**
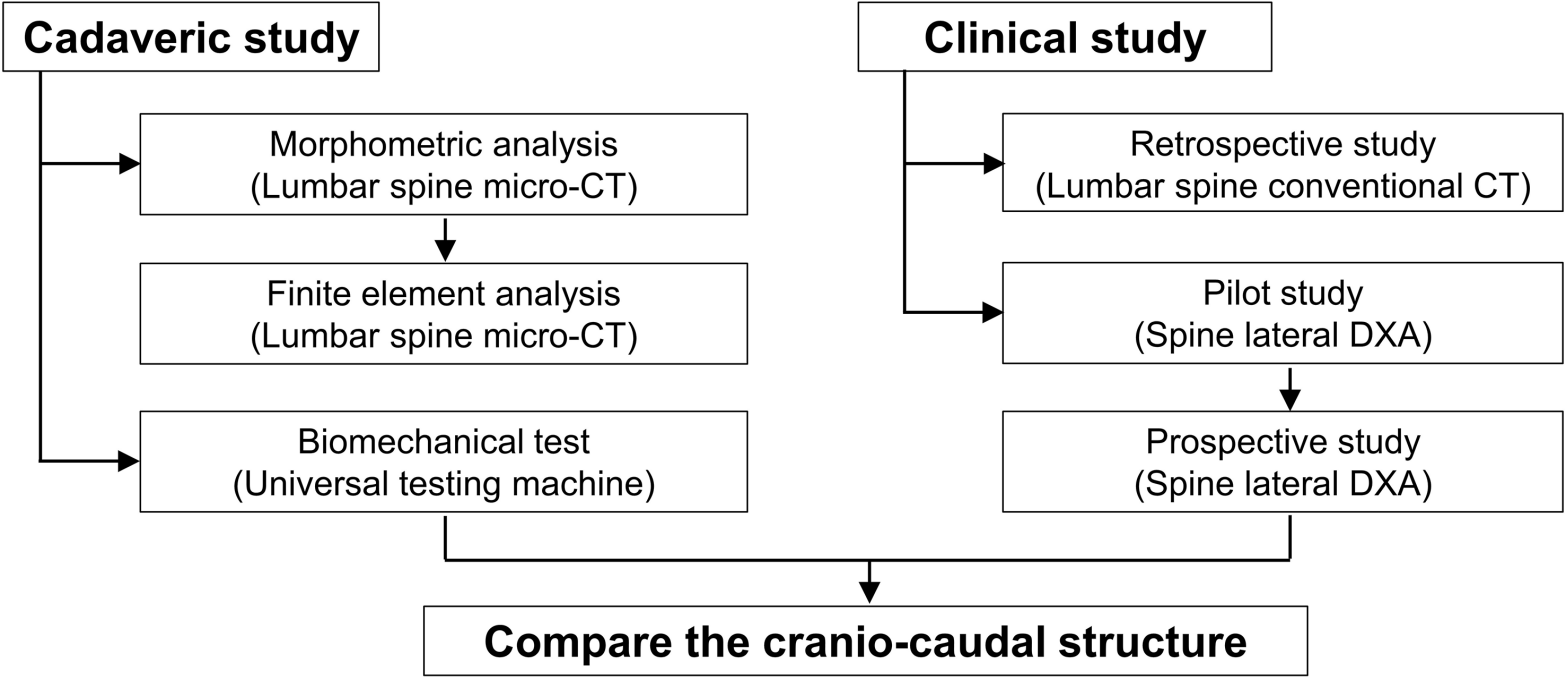
Study outline.

Initially, we hypothesized that the superior region would be more vulnerable than the inferior region of the individual vertebral body. To confirm this hypothesis, we harvested a lumbar vertebral body from a human cadaver. We then analyzed the (1) morphometric characteristics using micro-computed tomography (CT), (2) biomechanical strength using finite element analysis (FEA), and (3) biomechanical properties using a universal testing machine. Next, we retrospectively collected conventional CT images of the lumbar spine and analyzed the bone volume and Hounsfield unit (HU). Finally, we prospectively analyzed bone mineral density (BMD) using lateral spine DXA after the pilot study (**Figure 1**). The study design was approved by the local institutional review board (CHAMC 2017-06-014-012). The board waived the requirement for informed consent, and we encrypted all personal identifiers and analyzed the data anonymously.

### Cadaveric study: morphometric analysis using lumbar spine micro-CT

**Figure 2:**
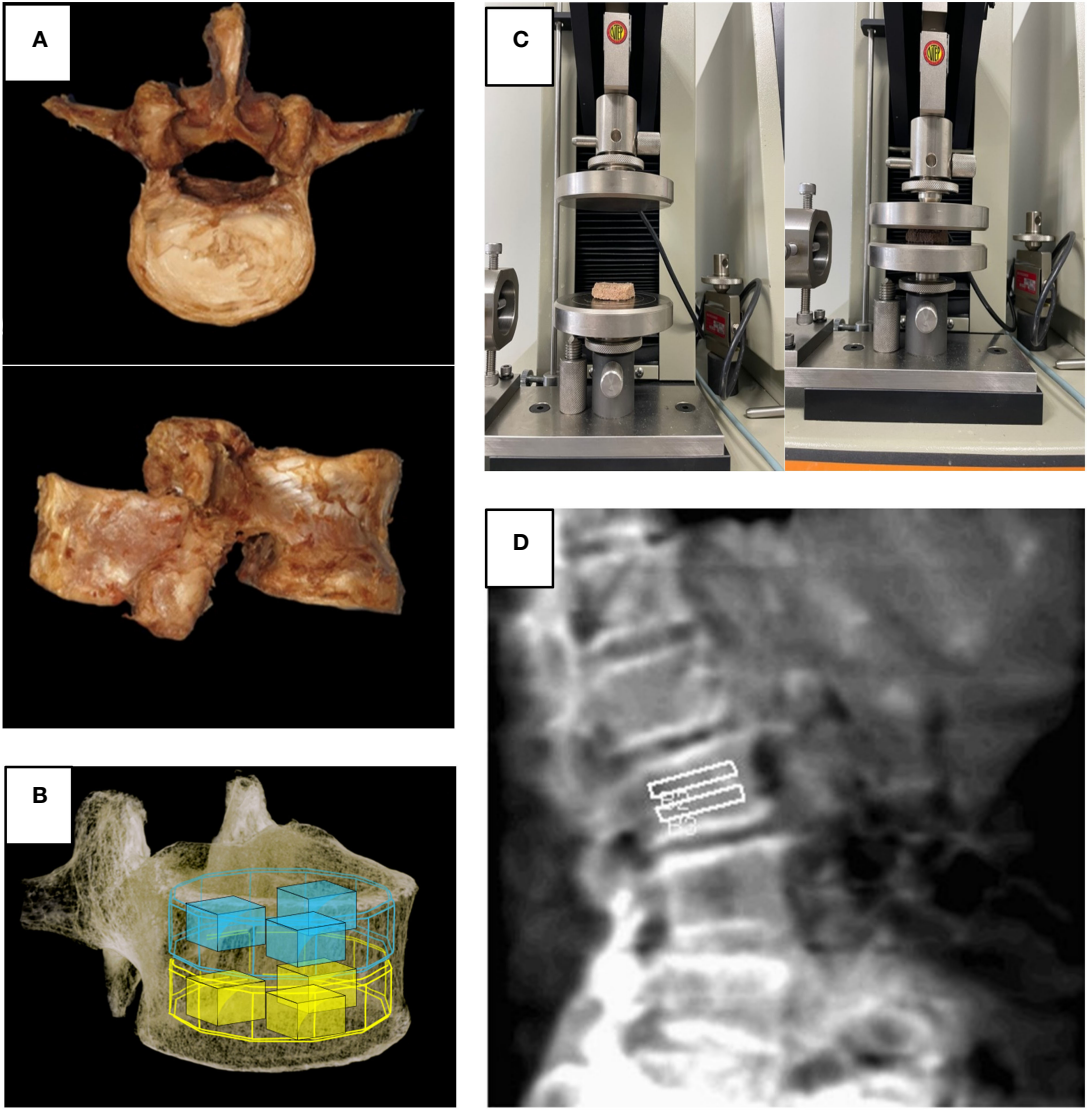
**(A)** Harvested lumbar spine for cadaveric study, **(B)** Volume of interest used in micro-CT and FEA, **(C)** Universal testing machine used in biomechanical test, **(D)** Region of interest for spine lateral DXA.

For the cadaveric study, five lumbar vertebrae were collected from three donors. The average age of the donors was 80.3 years, and they had no history of disease or trauma to the spine. Some lumbar vertebrae were excluded from the analysis because of compression fracture or severe spondylosis. The gross morphology of a single vertebral body after harvesting is shown in **Figure 2A**. The sample was fixed in formaldehyde. Because of the limited tube size, we removed the posterior column of the vertebra. Subsequently, these segments were scanned at a 50-µm resolution at a tube potential of 130 kV and a radiation source of 60 uA with 1.0-mm aluminum filter (SkyScan 1173, Bruker MicroCT N.V., Kontich, Belgium).

Subsequently, using the Feldkamp algorithm and technique, three-dimensional (3D) images were generated, and relevant image adjustments, such as ring artifact and beam hardening corrections and fine-tuning, were performed using the NRecon program (SkyScan 1173, Belgium). For optimal image contrast, the dynamic image range (contrast limits) was adjusted at 0–0.3 in units of attenuation coefficient and applied to all datasets. Next, the figures were reoriented on each 3D plane using the DataViewer software (SkyScan 1173, Belgium) to align the femur’s long axis parallel to the coronal and sagittal planes.

Morphometric parameters, such as percent bone volume (%), BMD (g/cm3), trabecular number (Tb.N, 1/mm), trabecular separation (Tb.Sp, mm), trabecular thickness (Tb.Th, mm), and bone surface ratio (BS/TV, %) were subsequently calculated from the binarized images using direct 3D techniques (marching cubes and sphere-fitting methods). All quantitative and structural characteristics are expressed using the terminology and units specified by the Histomorphometry Nomenclature Committee of the American Society for Bone and Mineral Research (7). We generated an 8 (width) x 8 (depth) x 10 (height) mm rectangular volume of interest (VOI) because the human vertebral body sample size was significantly large to be analyzed using one VOI. We subsequently placed three VOIs in the superior half of each vertebral body and three VOIs in the inferior half (a total of six VOIs in one vertebral body) and analyzed them part by part (**Figure 2B**).

### Cadaveric study: finite element analysis (FEA) using lumbar spine micro-CT

Micro-CT data were converted to Digital Imaging and Communications in Medicine images using the NRecon software. Subsequently, 3D mesh models were generated using the same VOI of the micro-CT analysis to investigate precise mechanical changes. Model meshing was performed using Mimics 3-Matics version 11.0 (Materialise, Leuven, Belgium), and FEA was performed with MIDAS-NFX using the same VOI as the micro-CT analysis (version 2021, MIDASIT, Korea).

### Cadaveric study: biomechanical test using universal testing machine

The area of each sample was determined before testing. The harvested specimens were tested for mechanical failure using a universal testing machine after each vertebral body was cut into superior and inferior halves (8). Between two solid plates, the specimens were crushed at a rate of 0.1 mm/s, and the load and displacement were recorded. The tensile strength was expressed in a unit area (kgf/mm^2^). The maximum load applied to each specimen was recorded (**Figure 2C**).

In this analysis, the trabecular bone model was assumed to be homogeneous and isotropic with linear elastic properties. The modulus of elasticity was 100 N/mm^3^, and Poisson’s ratio was 0.3. Convergence tests were performed to ensure sufficiently fine element discretization for displacement analysis. In the analysis process, 500 N of vertical forces was applied to the top of the spine cube model, and the bottom part was immobilized by setting the constraint condition. The von Mises stress and displacement parameters in the trabecular bone were reported. The VOI was set to be the same as that used in the micro-CT analysis.

### Clinical study: retrospective study using human lumbar spine CT

**Figure 3:**
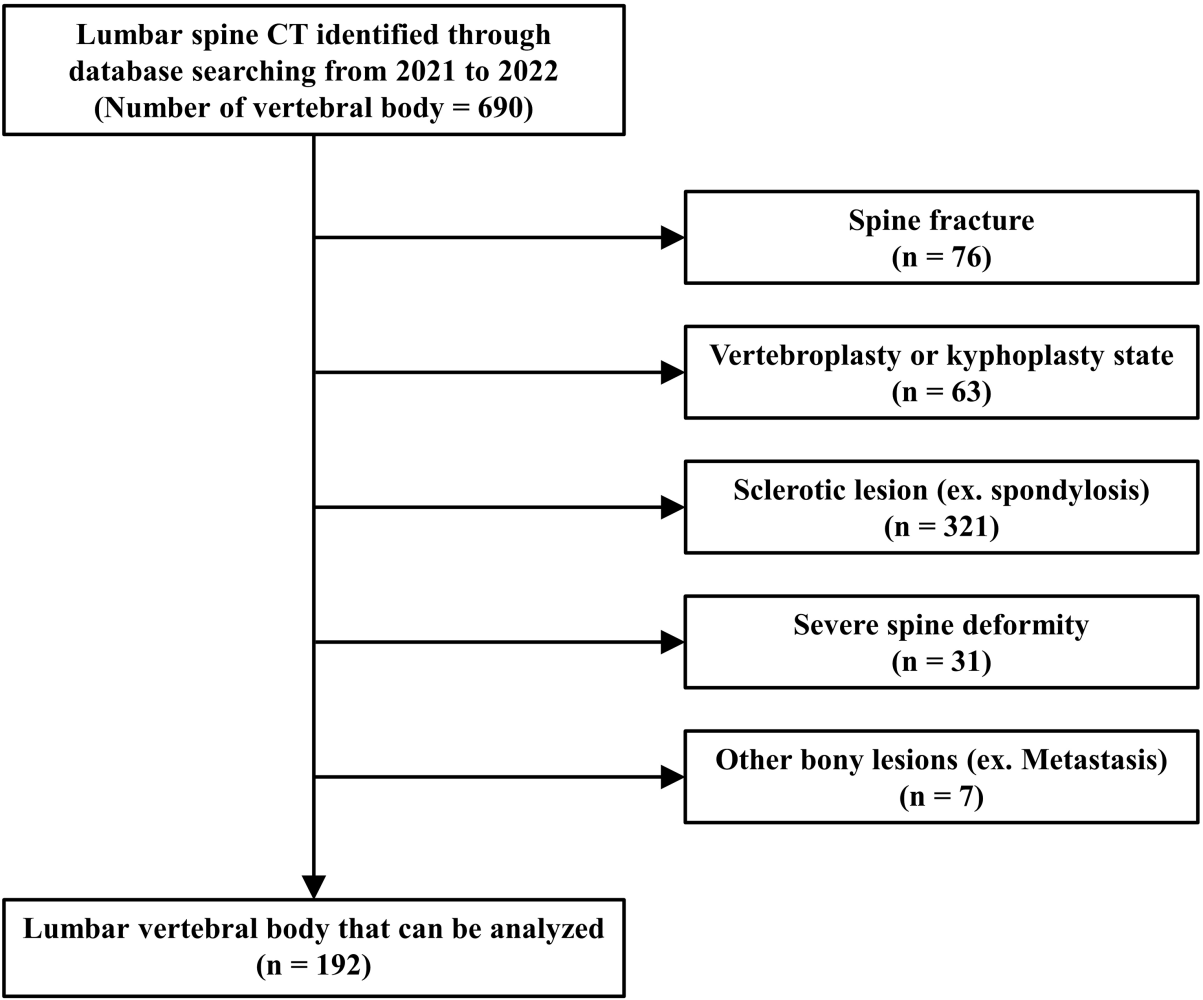
Study population of retrospective study using lumbar spine CT image.

We retrospectively investigated patients who underwent lumbar spine CT (Resolution CT, GE Healthcare, Milwaukee, Wisconsin, USA) from 2018 to 2021. CT images were acquired under the same conditions (120 kVp tube voltage, 23 noise index, 150–600 mA according to body thickness). In total, 115 participants (age range, 65–75 [mean age, 68.87] years [all participants were women]) were analyzed. The exclusion criteria were multiple spinal fractures, vertebroplasty with cement, sclerotic lesions of the spinal body (spondylosis), severe spinal deformity, and other bony lesions. According to these criteria, 192 vertebrae (thoracic spine 11th: 9, 12th: 32, lumbar spine 1st: 50, 2nd: 38, 3rd: 36, 4th: 27) out of 690 samples were analyzed (**Figure 3**). Bone volume (mm^3^) and BMD (HU) at the superior and inferior halves of the vertebral body were measured using SkyScan CT-Analyzer version 1.10. Regarding the spine CT analysis, because the spine CT had the low resolution, we analyzed the whole trabecular structure of each vertebral body after dividing superior and inferior halves.

### Clinical study: prospective studyusing spine lateral dual-energy X-ray absorptiometry

**Figure 4:**
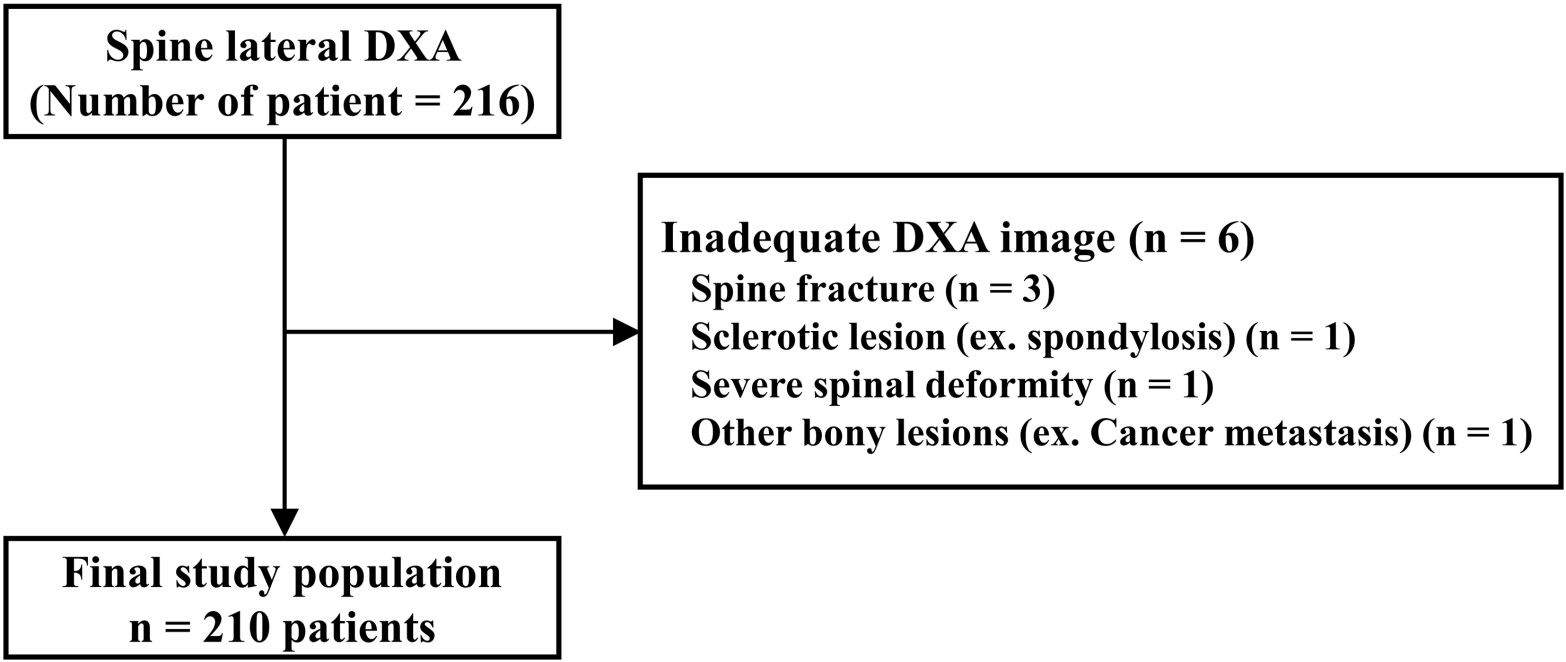
Study population of prospective study using lateral spine dual energy X-ray absorptiometry (DXA) image.

First, we calculated the sample size for this study. Throughout this study with 30 patients, we performed a sample size estimate using a two-sided t-test, with alpha and power levels of 0.05 and 0.80, respectively. These values were based on the results of preliminary studies. The assumed BMD (g/cm^2^) difference in the vertebral body was 4.98% between the inferior-half (0.622) and superior-half (0.591) groups. The minimum calculated sample size for each group was 617 (**Figure 4**).

Based on this result, we recruited postmenopausal women aged > 55 years who visited the CHA Hospital from January 2021 to September 2022. After applying the same exclusion criteria as in the retrospective study, 210 patients (630 vertebral bodies, L2, L3, L4) were enrolled for this study.

We measured the BMD of the vertebrae from L2 to L4 using DXA (Lunar Prodigy Advance, GE Medical Systems) in the lumbar lateral projection and analyzed its regional variations. We did not measure L1 because the L1 was overlapped by the rib cage in the lateral projection. The superior and inferior regions of each vertebral body were divided, and their evaluation was supervised by a senior radiologist (**Figure 2D**).

### Statistical analyses

Data are expressed as the mean ± standard deviation or range. Regional variations in the anatomical analysis and BMD between the superior and inferior regions of each vertebral body were explored using the paired t-test and Wilcoxon signed-rank test. Statistical software Statistical Package for the Social Sciences version 18.0 (IBM, Armonk, NY, USA) was used for all statistical analyses. Statistical significance was set at *P* < 0.05.

## Results

### Cadaveric study

**Figure 5:**
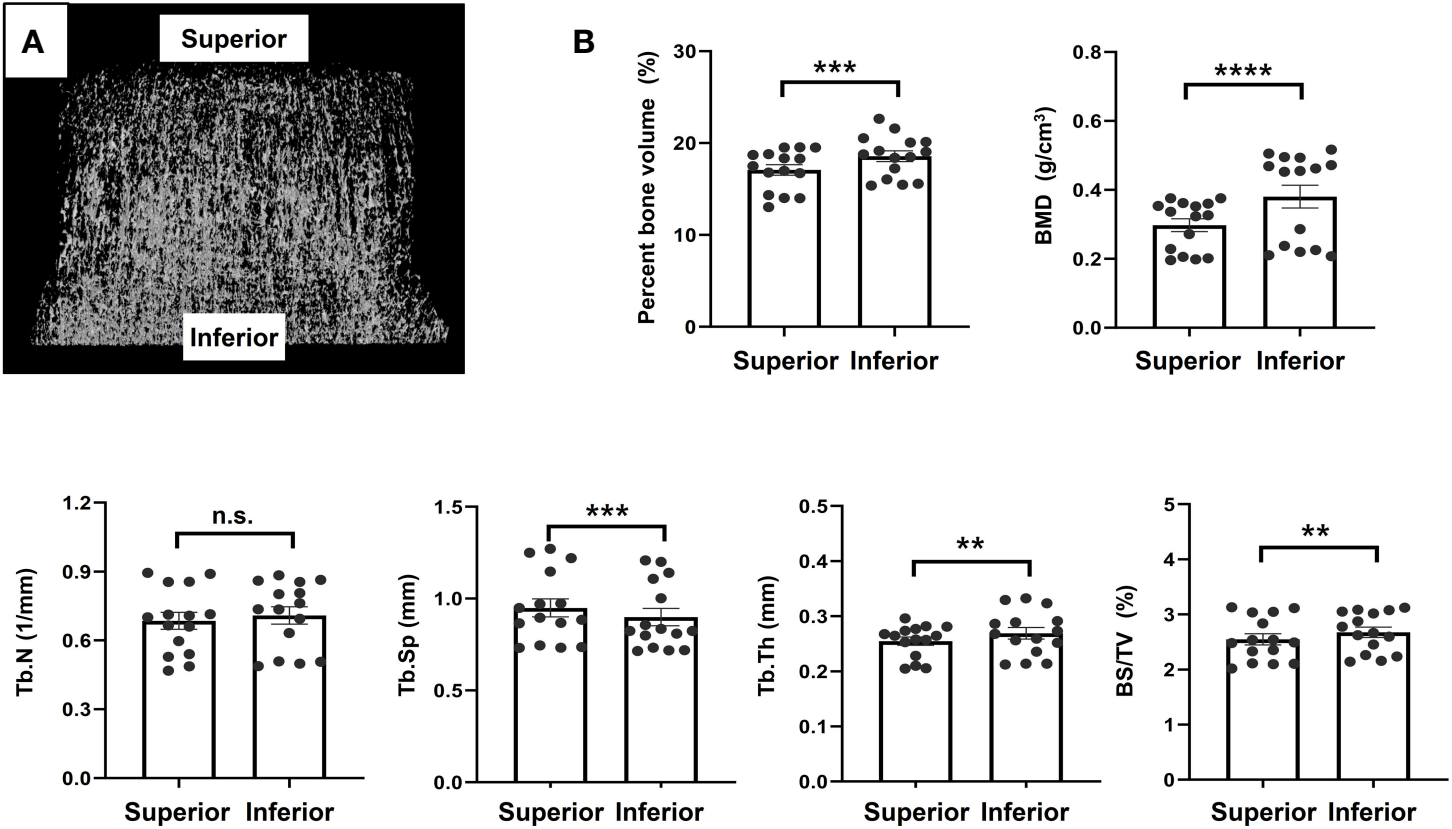
Micro-computed tomography analysis **(A)**. Representative image, **(B)**. Results of morphometric analyses using micro-computed tomography image. **p<0.01, ***p<0.001, ****p<0.0001. ns, non significant.

Micro-CT analysis revealed that the bone in the inferior region tended to be stronger than that in the superior region. There were statistically significant differences in the percent bone volume (17.07 ± 2.23 vs. 18.56 ± 2.26%, *P* = 0.002), BMD (0.30 ± 0.07 vs. 0.41 ± 0.18 g/cm^3^, *P* < 0.0001), Tb.Sp (0.93 ± 0.20 vs. 0.88 ± 0.20 mm, *P* = 0.0008), Tb.Th (0.25 ± 0.03 vs. 0.28 ± 0.05 mm, *P* = 0.01), and BS/TV (2.59 ± 0.42 mm vs. 2.75 ± 0.47%, *P* = 0.0017) between the inferior and superior regions. There was no significant difference in Tb.N between the two regions, although the inferior region had higher values than the superior region. The mean values of the bone morphometric parameters for the superior and inferior regions are shown in **Figure 5**.

**Figure 6:**
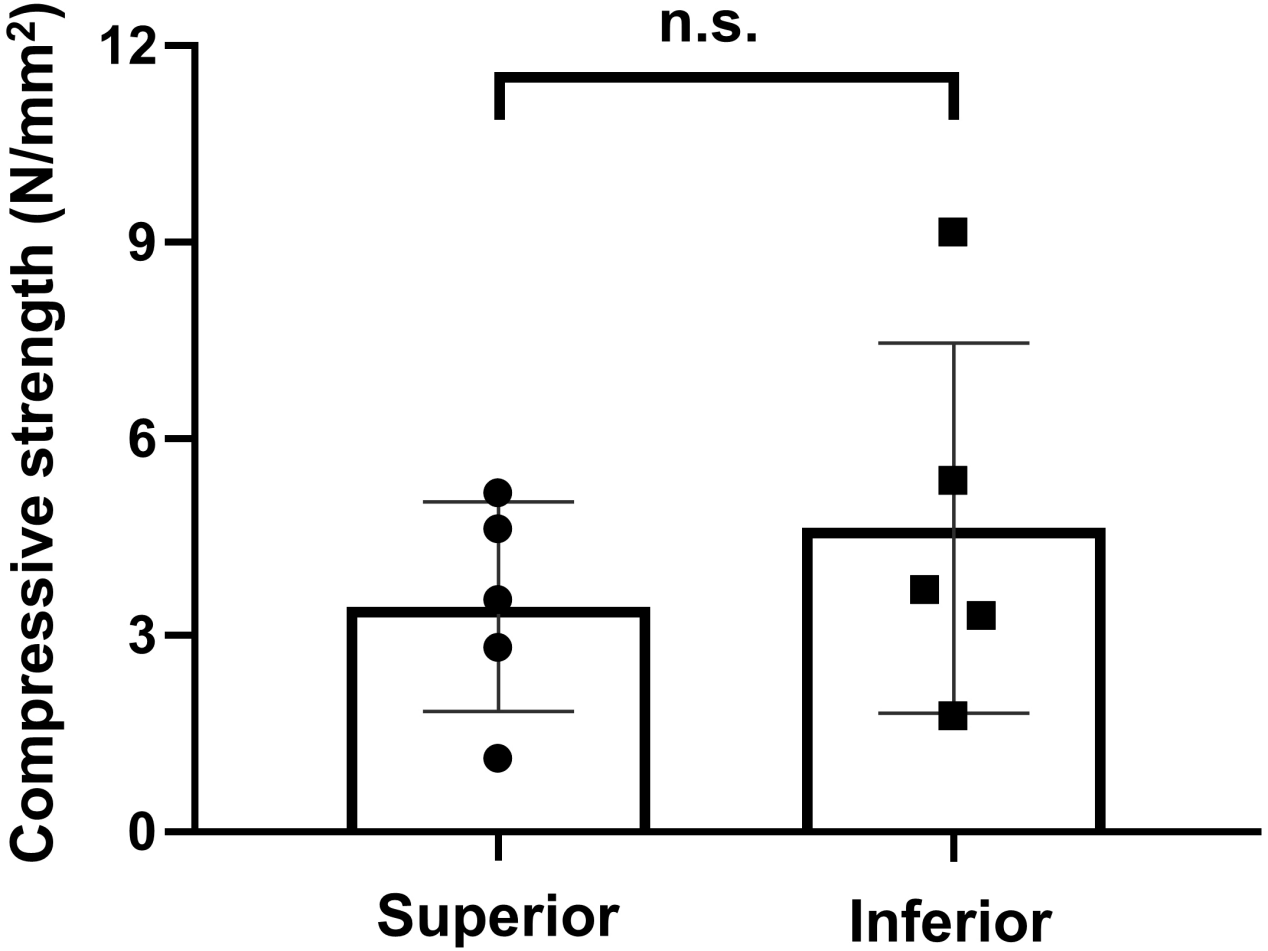
Result of biomechanical test using universal testing machine. ns, non significant.

For the ex vivo biomechanical test, compressive strength was applied using the same vertebral bodies used for the micro-CT analysis. The superior and inferior regions endured averages of 3.44 ± 1.6 and 4.64 ± 2.82 N/mm^2^, respectively. However, this difference was not statistically significant (*P* = 0.3125) (**Figure 6**).

**Figure 7:**
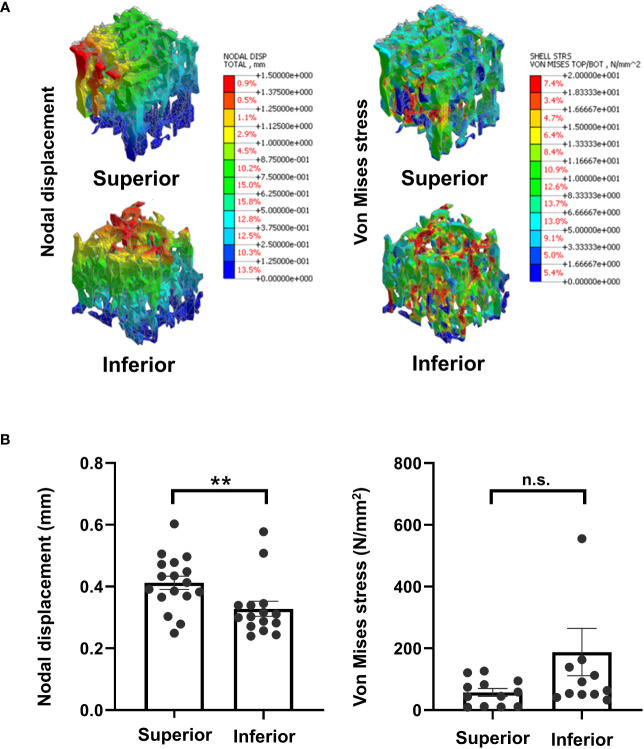
Finite element analysis **(A)** Representative image, **(B)** Results of morphometric analyses of finite element analysis. **P < 0.01, n.s.: Non-significant.

Next, FEA showed that the maximum von Mises stresses measured from the superior and inferior regions of the trabecular bone were 109.37 ± 73.65 and 161.06 ± 241.58 N/mm^2^ (*P* = 0.478), respectively. The average displacement of the trabecular bone model was significantly higher in the superior region compared with the inferior region (0.41 ± 0.09 and 0.32 ± 0.09 mm, respectively; *P* = 0.0067). The higher von Mises stress and lower displacement of the inferior regions of the vertebral body suggest that the inferior region has a larger contact area than the superior region under vertical force. This creates a stable structure, reducing stress and strain (**Figure 7**).

### Clinical study

**Figure 8:**
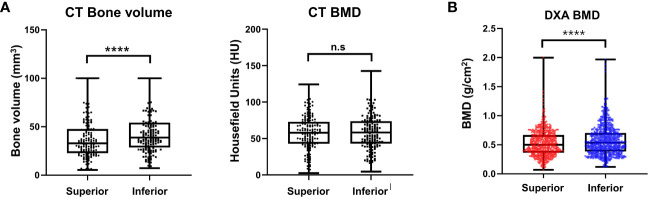
Result of morphometric difference by lumbar spine computed tomography **(A)** and dual energy X-ray absorptiometry **(B)**. ****P < 0.0001, n.s.: Non-significant.

In total, 192 vertebrae, from T11 to L4, were analyzed. The average bone volume was higher in the lower body than in the superior region at all vertebrae level, and the overall averages were 38.70 ± 22.86 and 44.41 ± 22.34 mm^3^ for the superior and inferior regions, respectively, showing a statistically significant difference (*P* < 0.0001). At each vertebral level, the HU of the superior and inferior regions showed no statistically significant difference, and the overall HU averages were similar at 59.33 ± 22 and 59.29 ± 22.83 units in the superior and inferior regions, respectively, which were not significantly different (*P* = 0.483). The results are presented in **Figure 8A**.

In this clinical study, 210 consecutive patients with a mean age of 70.6 (range, 55–85) years were included. A total of 210 patients with 630 vertebral bodies (L2–L4) were analyzed. Other vertebral body levels were removed from the analysis because of the shadowing of the ribs and pelvic bones on the lateral image. Differences in the regional BMD were noted between the superior and inferior trabecular bones. The mean BMDs of the superior and inferior regions of the vertebral bodies were 0.52 ± 0.22 and 0.57 ± 0.25 g/cm^2^ (*P* < 0.0001), respectively (**Figure 8B**).

## Discussion

In these cadaveric and clinical studies, we investigated the differences between the superior and inferior regions of the vertebral body. The results indicate that the superior region is more vulnerable than the inferior region. Bone volume, BMD, and various other trabecular parameters decreased in the superior region. A mechanical study confirmed that the limitation of force was higher in the superior region than in the inferior region. FEA analysis and clinical studies also supported our hypotheses; thus, the importance of lateral BMD, especially in the superior region, should be focused on and analyzed in the clinical field.

The vertebral structures were morphologically inhomogeneous within the body (9–12). Some studies on regional morphological variations in the vertebral body have been published. Zhao et al. reported that the cranial endplates were thinner than the caudal endplates by 14% and 11% on average in midsagittal and pedicle slices, respectively, and that the optical density of the trabecular bone adjacent to the endplate was 6% lower cranially than caudally. They concluded that endplate thickness was not the sole cause of failure of the superior region of the vertebral body and that the biomechanical properties of the endplate were dependent on the underlying trabecular bone (5).

Banse et al. reported transverse and vertical inhomogeneities in cancellous bone density inside the vertebral body using quantitative CT (pQCT) density analysis of the trabecular core (11). Hulme et al. divided cadaveric vertebral bodies into 10 regions of the cancellous bone to quantify regional variations in bone architecture parameters using micro-CT scanning. They concluded that the posterior regions of the vertebral body had greater bone volume, more connections, more trabeculae, lower Tb.Sp, and more plate-like isotropic structures than the corresponding anterior regions. Vertical inhomogeneity is observed only in the posterior regions. The posterior inferior region has higher bone quality than its corresponding superior region (9).

However, most of these studies were conducted using micro-CT and pQCT, which were not used in the current study, to clinically assess the bone quality of vertebral bodies. In addition, because some studies subdivided specimens into significantly several parts to evaluate intervertebral regional bone quality, the data from this study are limited for clinical application (13).

In this study, we simplified the division of the vertebral bodies into two regions (superior and inferior regions). Our cadaveric results were similar to those of previous studies, and the vertebral trabeculae of the superior region had greater quantitative and qualitative values than those of the inferior region. These structural variations were confirmed using DXA in postmenopausal women. Together with previous reports, the results of our study provide an explanation for why vertebral fractures are more common in the superior region of the vertebral body and more frequently involve the superior endplate.

We postulate that the better microstructure in the trabeculae of the inferior region of the vertebral bodies may be related to the anatomy of the vertebra and sagittal alignment of the thoracolumbar spine, where the superior region of the trabeculae connects to the posterior elements through a pair of pedicles. Compressive loads, which are the major causes of vertebral body failure in the thoracolumbar region, are partially transferred to the neural arch, which shields the superior region of the vertebra. However, the inferior region of the cortical shell does not have re-enforcement, lacks any posterior elements, and thus only has to rely on the underlying trabecular bone for mechanical endurance (14–17).

DXA in the anteroposterior projection is a conventional diagnostic method for the measurement of bone quality and includes the evaluation of cancellous bone, cortical shell, and posterior elements. However, it is influenced by sclerotic growth and osteophytes. Moreover, those posterior elements, such as both pedicles, facet joints, and lamina, do not contribute to the strength of the vertebral body (13, 18, 19). Therefore, it is not suitable for evaluating vertical inhomogeneity in the vertebral body. In contrast, using lateral DXA, the posterior elements that correspond to the mechanical strength of the vertebrae but increase their BMD may be removed from the region of interest setting (13, 19–21). Therefore, in our study, it was possible to confirm the vertical inhomogeneity of the vertebral body for real patients in the clinical study using lateral projection DXA.

To the best of our knowledge, this is the first study to combine microstructural variations of cadaveric vertebral trabeculae using micro-CT, which is a reliable and accurate approach to measure structural parameters and vertical variations of BMD in live patients using DXA. Therefore, we were able to overcome the limitations of previous single way studies.

Clinicians and researchers have developed methods to evaluate the quality of bone and hence fracture risk, such as the measurement of BMD determined through DXA, two-dimensional structural analysis of trabecular bone using scanning electron microscopy, and cortical bone thickness or geometry using CT and magnetic resonance imaging. BMD from conventional DXA imaging is the gold standard because it is noninvasive, easy to apply, and closely related to fracture risk (22–24). However, according to the 2000 National Institute of Health Consensus Statement, BMD is limited to predicting the occurrence of fractures because it reflects only approximately 70% of the bone’s strength (20, 25). In addition, BMD from conventional DXA image does not reflect the regional variability in the quality of the bone or vertebral geometry. Furthermore, pharmacological treatment strategies for osteoporosis have shown poor correlation between BMD and OVCF risk. Hence, the vertebral fracture risk may be better defined using more selective methods.

This study has some limitations that must be acknowledged. The asymmetry of the vertebral trabecular microstructure was related to age, disc degeneration, body mass index, and spine level. These factors can also alter the mechanical distribution patterns. However, our study analyzed all the samples together without considering these individual features. In addition, given the complicated stress distributions in the thoracolumbar spine that are influenced by posture and sagittal alignment, our results cannot fully explain the observed structural asymmetry. The structural asymmetry of the vertebral trabeculae appears to be a natural feature. Some patients did not have osteoporosis, and only postmenopausal women underwent clinical studies. In addition, we measured HU instead of BMD because we retrospectively collected the lumbar spine CT data. Although HU is not a BMD, we assumed that HU could be used for the analysis because HU was e correlated with BMD, and we compared the superior and inferior regions of the same vertebral body (the image was taken under the same condition) (26).

In conclusion, our study identifies poorer bone strength in the superior region of the vertebral body compared with the inferior region. In this context, lateral spine DXA may be useful for detecting bone quality in the superior region of the vertebral body.

## Data availability statement

The original contributions presented in the study are included in the article/supplementary material. Further inquiries can be directed to the corresponding author.

## Ethics statement

The studies involving humans were approved by CHA bundang medical center institutional review board. The studies were conducted in accordance with the local legislation and institutional requirements. The ethics committee/institutional review board waived the requiremen-t of written informed consent for participation from the participants or the participants’ legal guardians/next of kin because regarding the retrospective study, we only used the cadaver, which was scheduled for disposal after the medical students studied. Next, human CT images were analyzed retrospectively, so we could not get the informed consent. Finally, for the prospective study, lateral DXA image process was assumed as the routine procedure in this institute. Thanks.

## Author contributions

Study design: DS, SL. Data collection: YL, H-JA. Data analysis: J-WC, JO. Data interpretation: WA, JL. Drafting manuscript: YeL, CH, SL. All authors contributed to the article and approved the submitted version.
